# Widely Different Correlation Patterns Between Pairs of Adjacent Thalamic Neurons *In vivo*

**DOI:** 10.3389/fncir.2021.692923

**Published:** 2021-06-30

**Authors:** Anders Wahlbom, Hannes Mogensen, Henrik Jörntell

**Affiliations:** Neural Basis of Sensorimotor Control, Department of Experimental Medical Science, Lund University, Lund, Sweden

**Keywords:** thalamus, neurophysiology, adjacent neurons, correlation patterns, tactile

## Abstract

We have previously reported different spike firing correlation patterns among pairs of adjacent pyramidal neurons within the same layer of S1 cortex *in vivo*, which was argued to suggest that acquired synaptic weight modifications would tend to differentiate adjacent cortical neurons despite them having access to near-identical afferent inputs. Here we made simultaneous single-electrode loose patch-clamp recordings from 14 pairs of adjacent neurons in the lateral thalamus of the ketamine-xylazine anesthetized rat *in vivo* to study the correlation patterns in their spike firing. As the synapses on thalamic neurons are dominated by a high number of low weight cortical inputs, which would be expected to be shared for two adjacent neurons, and as far as thalamic neurons have homogenous membrane physiology and spike generation, they would be expected to have overall similar spike firing and therefore also correlation patterns. However, we find that across a variety of thalamic nuclei the correlation patterns between pairs of adjacent thalamic neurons vary widely. The findings suggest that the connectivity and cellular physiology of the thalamocortical circuitry, in contrast to what would be expected from a straightforward interpretation of corticothalamic maps and uniform intrinsic cellular neurophysiology, has been shaped by learning to the extent that each pair of thalamic neuron has a unique relationship in their spike firing activity.

## Introduction

Thalamic neurons have a tendency to produce oscillatory behavior and the relative synchronization of such oscillatory output can be directly read out using EEG (Steriade et al., 1991; Hirata and Castro-Alamancos, 2010). Oscillatory EEG waves can be detected in the relaxed state as well as in sleep (Sachdev et al., 2015). Under such circumstances, a critical factor is likely that thalamic neurons are induced to hyperpolarize, which leads to a relative unmasking of the intrinsic oscillatory behavior in the thalamic neurons (McCormick and Pape, 1990). Indeed, thalamic neurons are well-known for prominent intrinsic conductances that tend to endow them with pacemaker-like activity, “self-maintained oscillations” (Llinas and Jahnsen, 1982; Jahnsen and Llinas, 1984). When a sufficient number of thalamic projection neurons synchronize their oscillatory behavior, cortical synaptic activation becomes at least partly synchronized, and the resulting field potentials can become so large that they can even be measured on the outside of the skull as an EEG.

Although characterizations of preferred inherent resonant frequencies of thalamic neurons exist (Puil et al., 1994), it is not well described to what extent different thalamic projection neurons may differ with respect to their inherent resonant frequency. If different thalamic neurons have different densities of voltage-gated calcium channels in their membrane, or different densities of other reactive conductances such as the H-current (Yue and Huguenard, 2001), for example, they may as a consequence have different activation kinetics and thereby resonant frequencies (i.e., see Rongala et al., 2018 for example). Under the assumption that two neighboring thalamic neurons would be expected to receive more or less the same corticothalamic input (see below), if these two neurons also have the same resonant frequency, it would be expected that these two thalamic neurons would be nearly perfectly co-activated by the aggregated continual spontaneous background activity of the population of cortical neurons providing the corticothalamic synapses.

Thalamic neurons in part mediate sensory activation from the periphery and, to a larger extent, integrate cortical activation through the corticothalamic pathway (Turner and Salt, 1998). One possibility is that the exact inputs a thalamic neuron receives, i.e., the exact sensory and cortical neurons that provide it with synaptic input, is to a large extent determined by the anatomical position of the neuron within the thalamus. This notion is supported by an overall topographical organization of the sensory input to the thalamus, and a likewise overall topographical organization of the cortical input to the thalamus (Alitto and Usrey, 2003). An alternative possibility is that the afferent topography merely provides a background set of synaptic inputs, but that learning processes in the thalamic neurons in practice become the main determinant of which specific synaptic inputs each neuron receives.

Here, we wanted to explore these possibilities by making simultaneous recordings of pairs of thalamic neurons, which are located at essentially the same anatomical position. By comparing the correlation patterns between such pairs, we aimed to obtain information about the likelihood of the above alternative scenarios. We find that the correlation patterns differ extensively between pairs, regardless of location within the extent of the lateral thalamus, which suggests that adjacent thalamic neurons have different input connectivity and/or resonant oscillatory frequencies.

## Materials and Methods

### Surgical Procedures

Adult male Sprague-Dawley rats (*N* = 9, weight 326–371 g) were prepared and maintained under anesthesia using a mixture of ketamine and xylazine (20:1). The animals were first sedated using isoflurane (3% mixed with air for 30–60 s), followed by an i.p. injection (ketamine 40 mg/kg and xylazine 2 mg/kg) to induce anesthesia which was then maintained via a continuous intravenous infusion through the right femoral vein (approximately 5 mg/kg per hour of the ketamine component). The level of anesthesia was characterized by an absence of withdrawal reflexes to noxious pinches to the hind paw and the brain was exposed by a craniotomy. Following that, the level of anesthesia was additionally continuously monitored with an electrocorticogram (ECoG) electrode placed on the cortical surface dorsal to the thalamus, and further evaluated based on skin tone and respiration rate. The ECoG was characterized by irregular occurrences of sleep spindles, a sign of deep sleep (Niedermeyer and Da Silva, 2005). All animal experiment procedures in the present study were in accordance with institutional guidelines and were approved in advance by the Local Animal Ethics Committee of Lund, Sweden (permit ID M118-13).

### Recordings

Neurons were recorded using patch-clamp pipette electrodes extracellularly in the loose patch recording mode using the EPC 800 Patch Clamp Amplifier (HEKA, Lambrecht, Germany) without any applied filters. Patch-clamp pipettes were pulled from borosilicate glass capillaries to 6–15 MOhm using a Sutter Instruments (Novato, CA, USA) P-97 horizontal puller. The composition of the electrolyte solution in the patch pipettes was (in mM) potassium-gluconate (135), HEPES (10), KCl (6.0), Mg-ATP (2), EGTA (10). The solution was titrated to 7.35–7.40 using 1 M KOH.

The location of bregma was determined to be located at the point where the coronal and sagittal sutures crossed, and a flat elevation of the skull was ensured by placing bregma and lambda at the same relative height. All recording tracks were then aimed towards the thalamus according to stereotaxic coordinates defined by Paxinos and Watson (2006).

The electrode was advanced using a step motor until the dorsal part of the thalamus was reached, followed by a slower advancement of approximately 0.3–1 μm per second. At the same time stimulation electrodes located in the skin of digit 2 were repeatedly activated at 0.3 s intervals and evoked field potentials and neuron spikes were observed. Once two neurons were clearly observable at the same time and the amplitude of their action potentials seemed stable over time, a standard protocol of artificial electrotactile stimulation patterns (see below), separated by long intervals of spontaneous activity, applied to digit 2 was commenced. All data were digitized at 100 kHz using CED 1401 mk2 hardware and Spike2 software (Cambridge Electronics Devices, CED, Cambridge, United Kingdom).

### Tactile Afferent Stimulations

Four pairs of intracutaneous needle electrodes (made of stainless steel insect pins, size 000, diameter 0.25 mm, with etched tips) were inserted into predetermined sites in the skin on the volar side of digit 2 of the contralateral forepaw, in the same manner as in Oddo et al. (2017). These stimulation electrodes delivered 0.5 mA pulses lasting 0.14 ms. As a standard protocol, *via* this electrotactile interface, the animal was then episodically presented with eight different repeatable spatiotemporal patterns, which were reminiscent of the activation of primary afferents when touching objects with different curvatures, as described in Oddo et al. (2017). These stimulation patterns were delivered in a predetermined pseudo-random order, with 100 repetitions of each pattern. Each pattern lasted less than 350 ms (range 213–346 ms) and was followed by a period of 1.5 s without any stimulation.

### Post-processing

**Figure 1:**
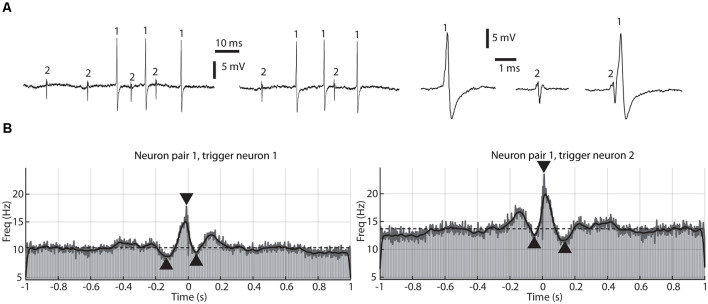
A paired recording of two adjacent thalamic neurons and their correlation patterns. **(A)** Sample raw traces, one at the beginning of the recording session (left), one at the end of the session (middle), about 20 min later, and magnified traces of the individual spikes, as well as a sample of coinciding spikes (right). Occurrences of the two separate spikes, recorded through the same patch pipette, are indicated by numerals (1 and 2, respectively). These two neurons correspond to pair #1 in Figure 2. **(B)** Correlation patterns between the two recorded neurons shown in **(A)**, one plot used neuron 1 as a trigger, and the other used neuron 2 as a trigger. The plots are peri-spike triggered histograms (PSpTH) with a bin width of 10 ms (light gray bars). Superimposed on the PSpTHs are Kernel Density Estimations of the spike-triggered activity (SpT-KDE). The gray line is an SpT-KDE with a short time constant (1 ms), and the black line an SpT-KDE with a longer time constant (10 ms). Horizontal dashed line indicates average baseline activity. Arrowheads indicate amplitudes and time points of the peak deflections, one for the pretrigger period, one for the peritrigger period, and one for the posttrigger period.

The recorded signal was imported from Spike2 to MATLAB (R2018b, The Mathworks, Inc.) where it was low-pass filtered using a moving average over 50 μs. Neural spikes were identified from the signal (Figure 1A) using tailored template matching routines with manually constructed templates. Each spike template was adapted to identify the same spike in all parts of the recording, as verified by visual inspection of a high number of random raw recording traces (visualized in time-voltage diagrams) in the beginning, the middle, and the end of the recording.

### Peri-spike Analysis

For each pair of recorded neurons, a peri-spike triggered time histogram (PSpTH) was created, with one neuron chosen as the triggering spike and the other as the responding spike. The PSpTH was constructed based on the relative timings between each trigger spike and all responding spikes occurring up to one second before and after the triggering spike (Figure 1B). The PSpTHs showed the spike-firing correlation pattern between the two recorded neurons in each pair. Next, two kernel density estimations (KDEs) of the activity of the responding spike in relation to the triggering spike were made (spike-triggered KDE, SpT-KDE), one with Gaussian kernels with a standard deviation of 10 ms and one with a standard deviation of 1 ms. The SpT-KDEs were used to identify parameters describing the shape of the spike-firing correlation patterns, with the 1 ms SpT-KDE being used to estimate time and magnitude of triggered activity with sharp peaks and the coarser 10 ms SpT-KDE being used for remaining parameters.

The analysis of the SpT-KDEs was based on the deflection from the baseline activity, with the baseline activity defined as the average of the 10 ms SpT-KDE from 1,800 ms to 500 ms before the triggering point and 500 ms to 1,800 ms after the triggering point.

From the SpT-KDEs we defined a peritrigger time window of activity deflection from the baseline around the triggering spike at time 0, a pretrigger time window activity deflection of 1 s before time 0 until the start of the peritrigger time window and a posttrigger time window of activity starting at the end of the peritrigger time window and lasting until 1 s after time 0 (Figure 1B). For each of these time windows, the timing and amplitude of the maximum frequency deflections from baseline activity were calculated.

The onset and ending of the pretrigger deflection were defined as the last continuous (>40 ms) depression of the 10 ms SpT-KDE below the baseline activity, before time 0. The pretrigger peak deflection was then defined as the net change in firing frequency between the baseline activity and the minimum value of the depression, divided by the baseline activity. The same procedure was used to define the posttrigger peak deflection, but instead, the first continuous deflection after time 0 was used. The peritrigger time window was then defined as the time between the end of the pretrigger time window and the posttrigger time window, and the peritrigger peak deflection was derived from the peak amplitude change from the baseline activity divided by the baseline activity, but now based on the 1 ms SpT-KDE, since these peaks could be too fast for the 10 ms SpT-KDE to capture.

Bootstrapping was used to resample the spike data 100 times for each pair and triggering neuron. A new sample of N responses was taken from the dataset using sampling with replacement, where N was the number of available samples and the sum of these samples was considered one bootstrapped response. The 95% confidence intervals for each parameter were then calculated.

**Figure 2:**
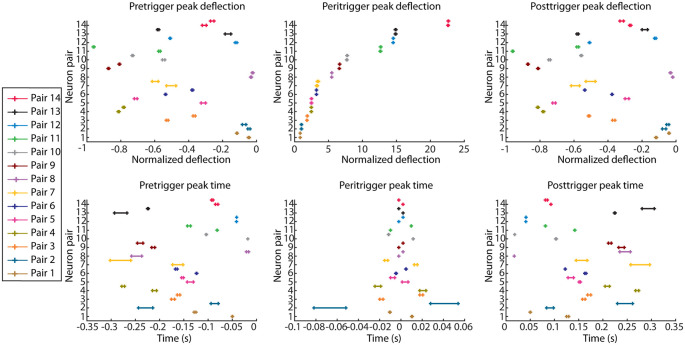
Parameter values quantifying the correlation patterns for all neuron pairs. Each correlation pattern was quantified based on the peak time and peak deflection amplitudes in the pretrigger, peritrigger, and posttrigger time periods. Neuron pairs were ordered based on their peritrigger peak deflection (top row, middle column). Note that each pair is represented two times, since each pair generates two correlation patterns, one for each triggering spike (Figure 1B). As the recorded data were bootstrapped for each pair of neurons, each parameter value is presented as a 95% confidence interval. Peak deflection values are the net changes in firing frequency from the baseline activity, divided by the baseline activity.

The same analysis was then repeated for each pair of recorded neurons, with the identity of the triggering and responding spike reversed, resulting in two PSpTHs, SpT-KDEs, and 95% confidence intervals for the calculated parameters for each pair of neurons (Figure 2).

The Pearson correlation coefficient was used to compare the 10 ms SpT-KDEs curves obtained when the triggering spike occurred during spontaneous and stimulated activity for all 14 neuron pairs.

### Firing Behavior Metrics

Four different metrics were calculated in order to quantify the firing behavior of each neuron. These were the same metrics as used in Wahlbom et al. (2021). In short, they were: (1) the average firing frequency; (2) the coefficient of variation (CV) of the interspike intervals (ISIs), calculated as the standard deviation of the ISIs divided by the mean ISI; (3) the average of neurons CV2 as calculated in Holt et al. (1996), which compares two adjacent ISIs; and (4) the logarithm of the firing regularity, calculated as in Mochizuki et al. (2016), where the firing regularity is the shape factor of a gamma distribution fitted to the ISI distribution. The CV2 of a neuron is calculated as

(1) $$CV2=\frac{2\left|ISIS_{i+1}-ISI_{i}\right|}{ISI_{i+1}+ISI_{i}}$$

where ISI_i_ represents the ith ISI. This results in a CV2 value for each pair of ISIs and was here presented as the average ISI of a neuron.

### ECoG Desynchronization Analysis

The EEG signal was detrended linearly with breakpoints every 10 s and then a Savitzky-Golay filter with a window length of 251 ms was used to low pass filter the signal. The amplitude envelope of the low pass filtered signal was calculated by taking the absolute value of the analytic signal using the Hilbert transformation. A segment was classified as desynchronized if it had a duration of at least 500 ms where the envelope amplitude was less than the mean envelope amplitude plus one standard deviation of the low pass filtered EEG signal.

The power spectral density below 8 Hz (representing delta 0.1–4 Hz and theta 4–7 waves) of a detected segment was compared to a segment of the same length as the detected segment, ending 50 ms before the start of the said detected segment, in order to see if suppression of these frequencies occurred during the postulated desynchronized segment. The raw signal of the detected segments was then inspected visually.

The Pearson correlation coefficient was used to compare the 10 ms SpT-KDEs curves obtained when the triggering spike occurred during synchronized and desynchronized activity for all 14 neuron pairs.

### Decoding Performance

A modified version of a previously published method (Oddo et al., 2017) was used in order to evaluate the neural response to the electrotactile stimulation. The method evaluates how well a neuron can differentiate between the eight different stimulation patterns presented using bootstrapping of neuronal data, principal component analysis (PCA), and k- nearest neighbor (kNN) classification. The result is then presented as the F1-score for the classification task, here called decoding performance. A more detailed description is described below.

(i) A Gaussian kernel with a standard deviation of 5 ms was used to convolve the spike trains evoked by the presented stimulation patterns into a continuous function, from the start of a stimulation up to 1,000 ms after stimulation start.
(ii) Half of the convolved responses were randomly chosen to be placed into a training set, and the other half of the responses were assigned to a test set. The two datasets were handled separately from the rest of this analysis.
(iii) Bootstrapping was used to resample the training dataset 200 times without any stimulation labels, and a new sample of N responses was taken from this population using sampling with replacement, where N was equal to the number of available responses. The sum of these responses was considered as one bootstrapped response. PCA was then used to determine the M principal components that were required to explain 95% of the variance in the bootstrapped data. The data in the training and test datasets were then grouped by stimulation pattern and bootstrapped separately in the same manner as above.
(iv) The scalar product between each bootstrapped response in the training and test data and the M principal component vectors was then calculated using the least-squares method. This was used to position each bootstrapped response in the M-dimensional space created by the principal component vectors.
(v) kNN classification was then used to decode the stimulation pattern for each bootstrapped response. The nine closest responses in the training set were determined for each bootstrapped response in the test set (nine nearest neighbors) by a Euclidian distance calculation in the M-dimensional space. The response was then classified as belonging to the same stimulation pattern that elicited the relative majority of the nine neighbors. The results of the classification procedure were then presented as a confusion matrix.
(vi) The above five steps were repeated 50 times, with each repetition having a new randomly assigned test and training set of responses. From the 50 repetitions, an average confusion matrix was obtained.

From the confusion matrix created by the classification procedure, the F1-score could be calculated as the harmonic mean of the matrix precision and recall.

### Histology

An estimation of the location of the recorded neuron pairs was made using their stereotaxic coordinates in relation to an atlas (Paxinos and Watson, 2006). An estimation of the precision of this method was calculated in Wahlbom et al. (2021), with a larger number of thalamic neuronal recordings, of which the neuron pairs used in this study were a subset. In short, the electrolyte solution used in the patch pipettes was mixed with Neurobiotin Tracer (Vector Laboratories) in order to stain the electrode tracks made when recording the neurons. The animals were transcardially perfused while under general anesthesia using 100 ml phosphate buffered saline (PBS) followed by 75 ml 4% paraformaldehyde (PFA) solution. The brains were then removed and placed in 4% PFA solution for 48–72 h for post-fixation and then transferred to PBS for storage. Before sectioning the brains, they were put in a 25% sucrose PBS solution for 48 h. A cryostat microtome was then used to cut the brain into 60 μm thick sections, which were then stained with Streptavidin conjugated to Alexa Fluor 488 (Molecular Probes Inc.). A confocal microscope was used to find any electrode tracts and their position was determined using the atlas by Paxinos and Watson (2006), which was then compared to the location according to the recorded stereotaxic coordinates. The estimated error of the stereotaxic coordinates was determined to be 0.24 ± 0.21 mm (mean ± standard deviation), and thus the estimated thalamic nuclei attributed to each neuron pair in this study can be assumed to be reasonably correct.

## Results

Using a previously described technique for dual neuronal recordings from single patch pipettes in the extracellular loose patch recording mode (Bengtsson and Jorntell, 2009; Bengtsson et al., 2013; Mogensen et al., 2019), we recorded a total of 14 neuron pairs/28 neurons distributed across the rostrocaudal extent of the lateral thalamus (Wahlbom et al., 2021). Figure 1A illustrates an example of a recording of a pair of thalamic neurons. Note the high signal-to-noise ratio, which made it possible to consistently identify the two unitary spikes even in cases where they coincided in time.

The main scope of this article was to identify and compare the patterns of correlation between the spikes of each thalamic cell pair, i.e., their cross-correlations. Figure 1B illustrates such cross-correlations. In each case, one of the spikes was used as the trigger point (triggering spike) and the distribution of the second spike (responding spike) in relation to the trigger point was plotted, for all occurrences of the triggering spike. The correlation pattern could be plotted as raw peri-spike time histograms (PSpTH) of the responding spike, or as spike-triggered kernel density estimators (SpT-KDE) of the same correlation. SpT-KDE resembles spike-triggered histograms, but instead of outputting the distributions across a set of time bins, it forms a continuous function. The continuous function consists of a convolution of Gaussian kernels centered over each spike time.

As shown in Figure 1B, the typical SpT-KDE profile contained a peak of activity surrounding the triggering spike around time 0 and a decrease in activity before and after the triggering spike. Gaussian kernels with a standard deviation of 10 ms were used to estimate the baseline activity and the points where the spiking activity deflected from the baseline activity before and after the triggering spike, respectively. To estimate the time and magnitude of the peri-spike peak activity, we instead used SpT-KDEs with a standard deviation of 1 ms for the analysis of the peak. A smaller standard deviation in the SpT-KDE results in a higher temporal resolution but an increased susceptibility to sampling errors, which is why this signal appears as a much noisier representation of the underlying PSpTH in Figure 1B. But, as can be seen in Figure 1B, the SpT-KDE with the higher temporal resolution (standard deviation of 1 ms) is better at capturing fast activity changes in the PSpTH around the triggering spike.

Although this peritrigger deflection was in the present material always positive, it was typically preceded and followed by troughs. We analyzed all three phases of the correlation patterns to compare different cell pairs. Figure 2 illustrates the peak time points and the peak changes in frequency (amplitude) for the pre-, peri-, and post-trigger events for all neuron pairs, with neuron pairs, sorted on basis of the amplitude of their peritrigger deflection. Each neuron pair is represented by its 95% confidence interval as calculated on the data produced by a bootstrapping procedure. Note that the values are dispersed widely along the x-axes and essentially non-overlapping between neurons at the 95% CI. Also, in cases of overlap between two neuron pairs for one parameter, they did not overlap across all parameters (Figure 2). This indicates that the correlation patterns were different between the pairs. Note that many of the correlation patterns were asymmetric, which for example is indicated by the variance in the peritrigger peak times and the fact that the pretrigger and posttrigger peak times occurred at different distances from time 0 within each combination of cell pair and triggering spike.

**Figure 3:**
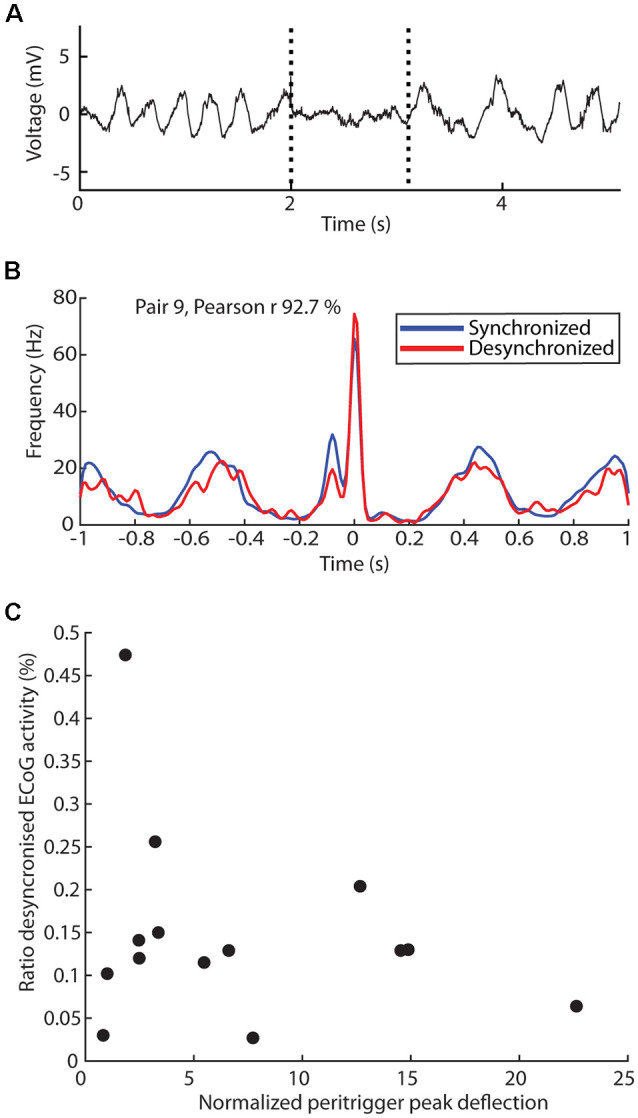
Dependency of the correlation patterns on ECoG state. **(A)** Sample raw trace of ECoG activity with transitions between synchronized to desynchronized state, and back again, indicated by dashed lines. **(B)** Comparison of the observed correlation patterns generated when the triggering spike occurs during synchronized (blue) and desynchronized (red) ECoG activity, with the Pearson correlation coefficient indicated. **(C)** Relationship between the mean peritrigger peak deflection in a thalamic neuron pair correlation and the time ratio spent in the desynchronized ECoG state during the recording of the pair.

**Figure 4:**
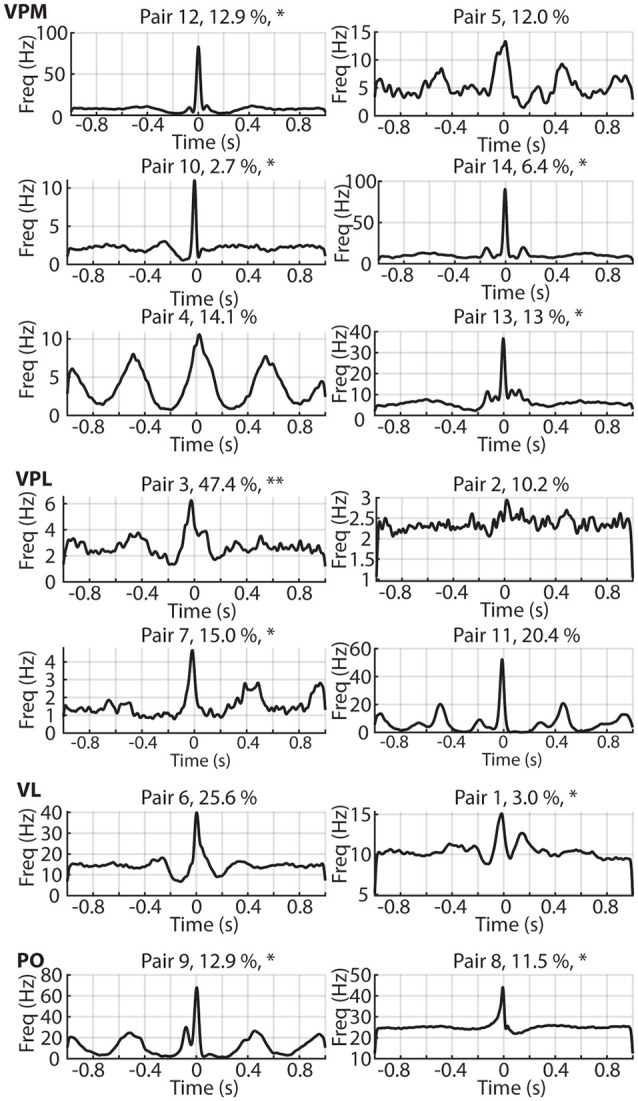
The correlation patterns for each recorded neuron pair. The time ratio spent in the desynchronized ECoG state is indicated as a percentage. Asterisks (*) are used to indicate the pairs of neurons which had one neuron which is defined as actively decoding the tactile afferent stimulation by having a decoding performance above our chance threshold level, two asterisks are used for pair 3 where both neurons surpassed this threshold (see Figure 5A). Also indicated are the estimated thalamic locations of each thalamic neuron pair. VPM, ventroposterior medial nucleus; VPL, ventroposterior lateral nucleus; VL, Ventrolateral nucleus; PO, Posterior complex.

As the thalamus is a key structure impacting the ECoG state (Steriade et al., 1991) and the present set of experiments was conducted under ketamine-xylazine anesthesia, which tends to affect the ECoG state (Soltesz and Deschenes, 1993), we also explored if differences in correlation patterns between neuron pairs could be due to persistent differences in ECoG state (Figures 3, 4). A surface electrode located on the cortex continually monitored the ECoG signal so that we could divide the recording sessions into time windows of synchronized and desynchronized ECoG activity (Figure 3A). Here we use the ratio of desynchronized ECoG activity of the total recording time [mean ± standard deviation of this ratio was 14.8% ± 11.1% (*N* = 14)].

We first explored whether the ECoG state would impact the correlation pattern within each neuron pair, illustrated for one neuron pair with a relatively pronounced periodicity in the correlation pattern (Figure 3B). Remarkably, the correlation pattern was not substantially impacted and the Pearson correlation coefficient for the two correlation patterns was as high as 92.7%. For the population of recorded pairs as a whole, the Pearson correlation coefficient was 74.3% ± 26.4% [range 6.2–99.2% (*N* = 28)]. Note, however, that in some of these cases the number of triggering spikes occurring during desynchronized activity was very low, thus creating a highly noisy correlation pattern. In principle, such noisiness could in itself cause a low Pearson correlation coefficient even though the underlying correlation pattern curves were essentially congruent. When we instead set an arbitrary limit that both neurons in a pair should have at least 400 spikes occurring during the desynchronized state for inclusion in this analysis, we instead obtained a Pearson coefficient of 89.7% ± −7.9% [range 74.3–99.2% (*N* = 14, i.e., 7 pairs)]. Hence, this analysis indicated an overall good congruence between the correlation patterns across ECoG states for a given pair of thalamic neurons. Furthermore, the ratio of time spent in the desynchronized state did not appear to impact the peak deflection amplitude (Figure 3C; except for one outlier the data points form an unstructured “cloud”, linear regression model *F*_(12)_ = 0.70, *p* = 0.42, *r*^2^ = 0.055) of the correlation patterns across the population of thalamic neuron pairs. Overall, this analysis suggested that the observed correlation patterns were not merely a by-product of the anesthesia-induced increased time spent in the synchronized ECoG state.

In order to determine if the intrinsic firing properties of the recorded neurons could explain the differences observed in their pairwise correlation patterns, we fitted a linear regression model to each of the six parameters quantifying the correlation patterns to the triggering neurons firing frequency, coefficient of variation of the interspike intervals (CV), CV2 which compares the relative difference between two adjacent interspike intervals (Holt et al., 1996) and the logarithm of the firing regularity measure as defined by Mochizuki et al. (2016), in total 24 fitted models. None of the parameters describing the firing behavior seemed to have any strong predictive value for the parameters describing the pairwise correlation patterns as they all had a low *r*^2^-values (*r*^2^ range 2.5 * 10^−4^ −0.16, *N* = 24).

We plotted all correlation patterns but could not find any predictive value of the ratio of desynchronized ECoG activity (indicated for each correlation pattern as a percentage) for the shape or magnitude of the correlations (Figure 4). Figure 4 also indicates the thalamic nucleus in which each neuron pair was recorded, but this anatomical location did not appear predictive of the correlation pattern either.

**Figure 5:**
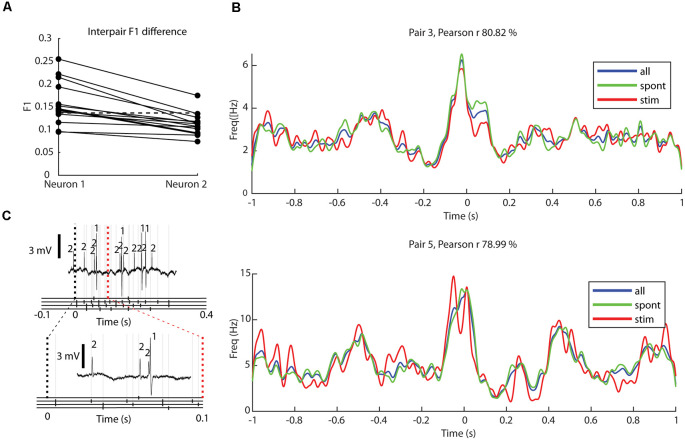
Decoding of tactile input patterns and impact of ongoing tactile input on correlation patterns. **(A)** The levels of decoding (F1 score) for eight specific tactile input patterns delivered to digit 2 for each neuron (*N* = 28). Neurons of the same pair are shown in separate columns and are connected with solid lines, where the neuron with the higher decoding is shown to the left. The dashed horizontal line indicates the limit where neurons were defined to be able to decode the tactile afferent input patterns (Wahlbom et al., 2021). **(B)** For two sample neuron pairs, superimposed correlation patterns for all recorded activity (blue), only activity occurring during ongoing tactile input patterns (red), and only activity excluding periods of ongoing tactile input patterns (spontaneous; green). For each sample neuron pair, the Pearson correlation coefficient between the latter two conditions (green and red curves) is indicated. **(C)** A sample raw trace showing the response of a neuron pair to one of the eight presented stimulation patterns (top) with a magnified trace below. Below each trace, the presented stimulation pattern is shown, with the occurrence of each electrical pulse shown with black markers in the corresponding stimulation channel, with light gray lines extending upwards.

The asterisks in Figure 4 indicate which neuron pairs contained one or two neurons which were found to decode tactile input patterns from digit 2 [such decoding occurs, surprisingly, across the entire extent of the thalamus where the definition of their locations was previously reported (Wahlbom et al., 2021)]. Figure 5 reports the decoding of such tactile inputs for all neuron pairs. The decoding performance, quantified as the F1 score, was 13.4% ± 4.4% across all neurons recorded. In our previous analysis of a larger set of thalamic neurons (Wahlbom et al., 2021) we defined a 14.1% decoding threshold limit above which a thalamic neuron with a reasonable level of certainty can be said to being capable of reporting on the quality of the tactile input to digit 2 using the present approach. As indicated in Figure 4, of the 28 neurons used in the present material (all of which were included in the larger dataset used in Wahlbom et al., 2021), only 10 neurons were above this threshold decoding level. Note that the neuron pair with the neuron having the highest decoding performance was the same neuron pair which was recorded at the highest ratio of desynchronized ECoG activity (Figure 3C, “outlier” in the ratio desynchronized axis). Although an anecdotal observation here, we previously have found that for cortical neurons outside the S1 paw region, the decoding performance increases during time windows with desynchronized ECoG activity (Enander et al., 2019). For adjacent neurons (Figure 5A), the difference in decoding level between the two neurons of the pair was 4.6% ± 3.0%, where the differences tended to be greater the higher the decoding level of at least one of the neurons of the pair.

Although the decoding levels of the tactile input were not so high, and hence the thalamic neurons of the present material were not so profoundly impacted by the tactile input from digit 2, we also controlled if the tactile input patterns (each lasting about 0.3 s, separated by about 1.5 s without any input) impacted the correlation patterns. For each neuron, we calculated one correlation pattern only including the time windows with ongoing tactile stimulation, and another correlation pattern only including the time windows excluding such stimulation (Figure 5B). To quantify possible differences, we calculated the Pearson correlation coefficient. Across the population, the correlation coefficient was 89.9% ± 14.7%. This high correlation value indicated that the ongoing stimulation did not produce substantially different correlation patterns.

## Discussion

Using single patch electrodes we recorded the spike firing activity of pairs of adjacent neurons (estimated to be located <10 μm apart) in different parts of the lateral thalamus and compared the correlation patterns between different pairs. We found that the correlation patterns were widely different between different pairs, that these patterns were often asymmetric, and that they did not change substantially with ongoing tactile stimulation patterns. Our findings suggest differences in the synaptic input connectivity rules and/or intrinsic properties across different pairs of adjacent thalamic neurons.

A central methodological issue was the reliability of the spike identification: how do we know that we did not miss spikes when they coincided in time? First, it should be pointed out that the loose patch recordings provided high signal-to-noise ratios of the recorded spike signals. This was a prerequisite for us to be able to separate spikes from two neurons located at a very short distance from each other. As we have previously shown, given enough signal-to-noise ratio of the two recorded spikes, it is theoretically possible to conduct this analysis without missing a single spike, not even when the two spikes coincide (Bengtsson and Jorntell, 2009; Bengtsson et al., 2013; Mogensen et al., 2019). Nevertheless, the problem with spike sorting is that it is hard to know if the analysis fails when spikes are systematically missed. Speaking against the possibility that a large number of spikes were missed when they coincided with the spikes of the other neuron of the pair is the fact that most of our correlation patterns were characterized by a high central peak around time 0.

Because of the high-resolution method, the two neurons of each pair were presumably located immediately adjacently to each other. It is of particular interest to study neurons located at the exact same anatomical spot because the overlap in the extent of their dendritic trees could be expected to be maximal. Hence, the overlap in available afferent inputs between the dendritic fields can be expected to be maximal for co-localized neurons. The synaptic inputs to thalamic neurons can be expected to be predominantly of corticothalamic origin even within primary sensory thalamic nuclei (Turner and Salt, 1998). Assuming that a canonical microcircuitry principle would be the main determinant of cortico-thalamocortical network connectivity (Alitto and Usrey, 2003), two adjacent thalamic neurons would then be expected to have essentially identical inputs and weights for those inputs. Indeed, the frequent presence of a central sharp peak in the correlation patterns would be in line with this hypothesis. Also, the presence of persistent oscillatory correlation patterns would be supportive of the notion of shared synaptic input combined with a similar refractoriness of spike generation, which would result in that also the intrinsic oscillatory properties would be similar between the two neurons of the pair. The correlation pattern of neuron pair#4 in Figure 4 would be in line with that interpretation. However, the presence of an asymmetry in the peritrigger peak and/or presence of asymmetry in pre- and post-trigger troughs (Figure 2) were instead supportive of the fact that the two adjacent neurons in a pair received different afferent inputs and/or differed in their intrinsic cellular properties. One such possible difference in intrinsic properties would be that one of the neurons in a pair would not be a thalamocortical projection neuron but rather an interneuron. This is unlikely, however, as interneurons make up a very small part of the neural population in the non-visual thalamic relay nuclei of the rat, as few as 1% (Arcelli et al., 1997). Furthermore, our results did not indicate any relationship between our four different firing behavior metrics (CV, CV2, firing regularity, and average firing frequency) and any of the parameters describing the correlation patterns.

Note that because the correlation patterns of the spontaneous activity did not significantly differ from the activity during ongoing cutaneous stimulation, it is likely that most of our correlation patterns were due to the corticothalamic inputs. This is further supported by the fact that no clear distinction could be observed in the correlation patterns between neuron pairs that responded to the cutaneous stimulation and those that did not, as indicated by their decoding performance. Other studies have instead focused on correlation patterns between thalamic cells as they are provided with strong sensory input, i.e., visual inputs to LGN cells (Yeh et al., 2009; Roman Roson et al., 2019). Due to the extreme skewness of synaptic weights for such primary sensory inputs in thalamic cells (Turner and Salt, 1998), one would expect thalamic cells to not be highly correlated during such sensory input even if they receive inputs from the same set of sensory afferents. This was indeed also found to be the case, and a potential explanation is that the individual thalamic neurons have learned the specific synaptic weights for the retinal inputs, such that they become differentially skewed across the population of thalamic neurons.

The fact that these asymmetries in the correlation patterns differed between different pairs indicates that the relative differences in afferent inputs between the two neurons varied between neuron pairs and/or that their intrinsic threshold and/or refractoriness of spike generation differed in properties. Note that assuming that the activity of the corticothalamic inputs to different pairs systematically differ in their spatiotemporal structure, due to differences in localization within the thalamus, could not alone suffice to explain the presently observed differences in correlation patterns. Hence, the present results indicate that the afferent inputs to adjacent thalamic neurons are to at least some extent defined by learning, and/or that they, as a rule, are at least somewhat different in their intrinsic neurophysiological properties, for example, preferred intrinsic oscillatory frequency (Puil et al., 1994). Naturally, systematic differences in the anatomically defined corticothalamic inputs to two adjacent neurons are also a possibility although such a phenomenon remains to be shown. Either of these factors could cause even two adjacent thalamic neurons to respond quite differently, even though they share the same population of anatomically available synaptic inputs, compared to other thalamic pairs, which share other corticothalamic inputs. Hence, our results suggest that across the thalamus there are pair-specific differences in synaptic weights or in intrinsic cellular properties for adjacent thalamic neurons. Such fine-grain and detailed differences could possibly be acquired through learning.

## Data Availability Statement

The data and meta-data used in the study is available at: https://doi.org/10.6084/m9.figshare.c.5463828.

## Ethics Statement

The animal study was reviewed and approved in advance by the Local Animal Ethics Committee of Lund, Sweden (permit ID M118-13).

## Author Contributions

AW and HJ designed the experiments. AW performed all experiments. AW, HM, and HJ designed and performed the analysis and wrote the manuscript. All authors contributed to the article and approved the submitted version.

## Conflict of Interest

The authors declare that the research was conducted in the absence of any commercial or financial relationships that could be construed as a potential conflict of interest.
